# Frailty in Primary Care: Validation of the simplified Zulfiqar Frailty Scale (sZFS)

**DOI:** 10.3390/medicines8090051

**Published:** 2021-09-03

**Authors:** Abrar-Ahmad Zulfiqar

**Affiliations:** Service de Médecine Interne, Diabète et Maladies Métaboliques de la Clinique Médicale B, Hôpitaux Universitaires de Strasbourg et Equipe EA 3072 “Mitochondrie, Stress Oxydant et Protection Musculaire”, Faculté de Médecine, Université de Strasbourg, 67000 Strasbourg, France; abzulfiqar@gmail.com

**Keywords:** frailty syndrome, primary care, sZFS tool, GFST scale

## Abstract

Introduction: Frailty scales are used very rarely by general practitioners as they are time consuming and are not well-adapted to current needs. Thus, we have designed with general practitioners a new scale for the early and rapid detection of frailty syndrome, called the simplified Zulfiqar Frailty Scale (sZFS). Patients and methods: This scale was tested in two general medicine practices in Normandy (France) for a total of six months and compared to the GFST tool “The Gerontopole Frailty Screening Tool”. Only patients who were over 65 years old with an ADL ≥ 4/6 were included. Results: 107 were patients included in the general medicine practice, with an average age of 74 years. The sZFS questionnaire has a shorter administration time than the GFST questionnaire (*p* < 0.001). Its sensitivity is of 93%, and its specificity is 58%. Its positive predictive value is 57%, and its negative predictive value is 93%. The area under the curve of the sZFS scale is 0.83 [0.76; 0.91] (IC95%). Conclusion: Our frailty screening scale is simple, relevant, and quick.

## 1. Introduction

Preventing dependency is a public health objective. Frailty can be used to predict the risk of dependency, falling, hospitalization, and death. General practitioners would be the best choice of health care professional for identifying frailty, but it is hard to do this in current practice with validated tools. 

There is no consensus regarding frailty diagnostic criteria. The prevalence of frailty depends on the tool used. In the European SHARE study, the prevalence of frailty varied from 6% to 43% depending on the eight tools used [1]. These tools were validated by international cohort studies for diagnosing frailty, but appear difficult to use in general medical practice. Therefore, we have developed a tool for identifying frailty in general medicine for independent subjects over 65 years old that is intended to be quick and easy to use. It takes into account various factors related to frailty risk (social, cognitive, nutritional, falls, and iatrogenic).

The Fried Scale is widely known [2], but its inclusion in measurements is not routinely used for patient assessment. The Frailty Index (FI) of Cumulative Deficits (FI-CD) was proposed by Rockwood and Mitninski. It is well validated and has a higher predictive ability of adverse clinical events than other frailty measurements in both hospital and community settings [3,4], but it has some limitations and is time consuming. There is also a Frailty Index derived from the Comprehensive Geriatric Assessment (CGA). It is used as a clinical standard for frailty assessment and has been found to be highly correlated with the FI-CD [5]. It is also time consuming. 

The Gerontopole Frailty Screening Tool (GFST) consists of two parts: a questionnaire performed first, and the clinician’s judgement of frailty status [6,7]. A limitation of this scale is that it does not provide specific guidance for clinicians regarding the identification of frailty. Moreover, most of the items are subjective.

We therefore developed a frailty screening tool for use in primary care, referred to as the Zulfiqar Frailty Scale (ZFS). This scale was tested in a general practitioner’s office for six months in Plancoët, France. This first study was published in Medcines MDPI [8]. The difference with the original ZFS scale [8] is that the simplified scale has five questions with only one social question, instead of two in the original scale. Indeed, the item “presence of caregivers” was not retained in this simplified scale, as this explained by the realization of an autonomy assessment with the ADL scale, which by a score ≥ 4/6 indicates a certain autonomy of the subject included.

The main purpose of this second study was to evaluate the ability of the fast-acting “simplified Zulfiqar frailty scale” (sZFS) tool to detect frailty among a group of elderly patients who are monitored by a general practitioner, in comparison with The Gerontopole Frailty Screening Tool (GFST).

## 2. Material and Methods

### 2.1. Study Type

Prospective and observational study conducted in two general practices in the Normandy region of France. 

Patients were selected to participate in the study over a period of six months, between November 2017 and April 2018. 

### 2.2. Study Population

Our study population was made up of patients aged 65 or older who were monitored by a general practitioner and had an ADL (Activities of Daily Living) score of 4/6 or higher. Patients who did not provide their verbal consent during the introductory phase of the study, were under 65 years of age, had an ADL score of less than 4/6, or lived in nursing homes were excluded from the study.

### 2.3. Study Parameters

#### 2.3.1. Characteristics of the Population 

The data collected were gender, age, the Activity (Katz Index of ADL [9]) and Instrumental (Lawton Index of IADL [10]) of daily living score, the medical comorbidities, the Charlson comorbidity index [11], and the weight. 

#### 2.3.2. Frailty Screening with the “simplified Zulfiqar Frailty Scale” (sZFS) Tool

The score was calculated by way of five indicators that measured the main functions of an elderly person [2,12,13,14] in terms of their geriatric relevance as defined by the scientific literature. A point was assigned for each positive indicator (maximum score = 5).

Each item was selected based on its quick completion time and simplicity so that prior training was not needed. The aim of our tool is to identify five elements considered to be significant according to the literature. See Table 1 the questionnaire of the simplified ZFS tool. 

These variables are significantly and independently associated with an increased risk of occurrence of negative events in terms of morbidity and mortality [15,16]. The difference with the original ZFS scale [8] is that the simplified scale has five questions with only one social question, instead of two in the original scale. See Table 1 for the description of the sZFS.

Each item, if present, accounts for one point (maximum score: 5). An elderly subject is considered as “not frail” with a score of 0/5, “pre-frail” with a score between 1/2 and 2/5, and “frail” with a score ≥ 3/5.

#### 2.3.3. Frailty Screening with the GFST 

The Gerontopole Frailty Screening Tool (GFST) comprises two parts: a questionnaire is performed first, followed by a clinician’s judgement of frailty status [6,7,17].

#### 2.3.4. Statistics

The “sZFS” score was assessed in terms of sensitivity, specificity, positive and negative predictive values, and the area under the ROC curve, using the GFST scale. A Pearson correlation matrix was used to evaluate discrepancies between the total scores and the items of each score. A paired two-sample t-test was used to compare the time it took to administer the two questionnaires. All the analyses were performed with R 3.6.1 software with an alpha risk set at 5%.

The study has been registered with the CNIL “National Commission on Informatics and Liberty”. 

Ethic consideration: Written consent from patients included were obtained. Internal Department Ethics Committee approved this paper for publication (N°15-01-18).

## 3. Results

### 3.1. Description of the Population

107 patients over 65 years old were included. No refusals were noted. The characteristics of the population included are detailed in Table 2.

### 3.2. Frailty Measured by GFST et sZFS Scales

Table 3 presents the results by item of the GFST frailty score. The results show that elderly subjects were considered frail according to the GFST scale for 34.6% of the population included. See Table 3.

Table 4 presents the results by item of the sZFS frailty score. The results show that the elderly subjects were considered frail according to the sZFS scale for 60.7% of the population included. See Table 4.

### 3.3. Main Objective

#### 3.3.1. Correlation between the GFST and sZFS

The Pearson correlation coefficient and its 95% confidence interval is 0.81 [0.73—0.86], (*p* < 0.001). See Table 5 details about Pearson correlation.

#### 3.3.2. Study of Sensitivity, Specificity, Positive Predictive Value and Negative Predictive Value

The sensibility was at 93%, while the specificity was at 58%. The positive predictive value (PPV) was at 57% and the negative predictive value at 93%. See Table 6.

**Figure 1:**
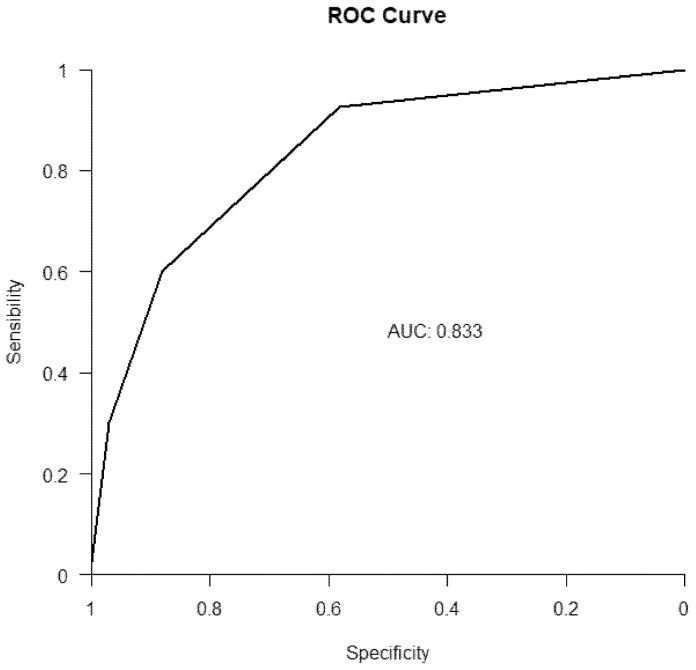
ROC curve, “sZFS” vs. GFST.

The area under the curve of the sZFS scale is 0.83 [0.76; 0.91] (IC95%). Note: the sZFS seems to be a relevant tool, it offers a good AUC. See Figure 1.

## 4. Scales Administration Time

The mean difference in administration of the scales is 9.5 s, CI [7.2; 11.8]. (See Table 7)

The mean time of administration of the sZFS questionnaire was statistically different from the mean time of administration of the GFST questionnaire (*p* < 0.001). The sZFS questionnaire has a shorter administration time than the GFST questionnaire.

Its use in primary care seems possible.

## 5. Discussion

The issue of screening for frailty among the elderly people is growing with the demographic changes we are experiencing today and is set to increase in the coming years and decades. One of the major roles in screening for frailty is played by the general practitioner, who is at the crossroads of the latter, due to the frequent and repeated contact that he or she maintains with the elderly patient in his or her monitoring role, and the influence that he or she can have on the future of the patient in his or her role of coordinating care and management with the various other health, medical, paramedical, and social players. The psycho-medico-social reflection that comes from this frailty has given rise in recent years to different scales or different screening scores, with the aim of providing optimum care for these elderly people, and particularly frail elderly people. However, very few frailty scales are used by general practitioners as they are time consuming and are not well-adapted. We have therefore created this rapid screening scale, taking into account the clinical, psychological, and social dimension of the patient, trying to adapt it as well as possible to general medicine. This meant that it had to be simple, efficient, quick to implement, sensitive, and with a high negative predictive value.

Our first study published in Medicines MDPI [8] concerned only older subjects over 75 years old. With this work, we decided to lower the age of inclusion to 65 years in order to have a heterogeneity of the frailty profiles ranging from the non-frail and pre-frail character in a not insignificant proportion to the frail subjects that we see more frequently in very older patients and the results in terms of the proportion of frail subjects confirm this between the two studies.

Our scale is intended to be very simple to pass on to general practitioners. A screening tool must be simple, quick, with good sensitivity and a good negative predictive value, which is the case for our frailty screening scale. Our frailty scale has several advantages. Indeed, it does not require prior training of the medical staff, nor does it require a long time to be administered, making a medical consultation, which is already quite long when it is dedicated to elderly subjects, more time consuming. In France, the usual duration of a consultation in general medicine is 15–16 min [18]. With our frailty detection scale, the time taken to complete the procedure is less than 2 mins.

Unlike the Fried scale [2], our scale does not require any additional equipment such as a dynamometer for measuring isometric contraction. This is a real advantage in the context of large-scale screening. The advantage of our scale compared to the GFST “Gerontopole_Frailty_Screening_Tool” [6,7,17] scale, for example, also lies in a better objectivity in the nature of the items selected. Indeed, the GFST scale contains more subjective questions whereas our scale would have the advantage of being more objective while being as simple to administer. In addition, we propose a rating, which allows the general practitioner to be guided.

Our goal was to create a rapid frailty screening scale that would be useful for general practitioners. The purpose of our scale is the early detection of frail elderly people, helping to delay the loss of autonomy. The value of systematic screening for frailty in the general practice requires large-scale prospective studies. Adapted physical activity, nutritional management, and diagnosis of underlying pathologies are the main axes of interventions.

We recognize weaknesses in our work, particularly on the number of subjects included which remain limited and weak. In addition, we recognize a high rate of false positives. It would be useful to continue the work on a larger workforce, on several general medicine practices and to be able to study the real agreement between our rapid frailty screening scale and a comprehensive geriatric assessment (CGA) performed by geriatricians.

## 6. Conclusions

To be validated, our scale must be tested further in other general practices by recruiting a wider range of participants. Furthermore, the reproducibility and ability of the scale to predict potentially dangerous situations (morbidity-mortality, hospitalizations and passage to the ER) must be developed and tested on elderly patients, which will take place in the upcoming weeks and months. A study is underway in the Poitiers region, France, with the use of our scale and a comparison with the Fried scale, in two general medicine offices, and another one in Champagne Ardennes region in 12 general medicine offices, with comparison between our scale and Fried scale. These results will be communicated soon.

## Figures and Tables

**Table 1:** The simplified Zulfiqar Frailty Scale (sZFS).

The simplified Zulfiqar Frailty Scale (sZFS)		
Nutritional status: Is there a weight loss greater than or equal to 5% in 6 months?	Yes	No
Balance/falls: Monopod support test <5 s?	Yes	No
Social isolation: Does the person live alone at home?	Yes	No
Cognitive functions: Does the person complain of memory problems?	Yes	No
Polymedicine: Does the person have prescriptions for more than 5 therapeutic classes on his/her prescription history for less than 6 months?	Yes	No

**Table 2:** Population characteristics.

	*n* = 107
Age, m (sd)	74 (7)
Gender	
Female	64 (59.8)
Male	43 (40.2)
Weight (kgs), m (sd)	70.8 (13.4)
Medical comorbidities, m (sd)	2.7 (1.3)
Medical comorbidities (%)	
Cardiovascular	78 (72.9)
Pulmonary	19 (17.8)
Renal	18 (16.8)
Gastrointestinal	40 (37.4)
Endocrine	40 (37.4)
Neurological	14 (13.1)
Psychiatric	22 (20.6)
Osteoarticular	50 (46.7)
Oncological	13 (12.1)
Therapeutic classes, m (sd)	4.2 (2.2)
Medication, m (sd)	5.1 (2.9)
Charlson comorbidities index, m (sd)	4.38 (1.99)
ADL (/6), m (sd)	5.87 (0.34)
IADL (/4), m (sd)	7.65 (0.85)

ADL: Activity Daily Living; IADL: Instrumental Activity Daily Living.

**Table 3:** Frailty measured by GFST scale.

	*n* = 107
GFST items	
Does your patient live alone?	34 (31.8)
Has your patient involuntarily lost weight in the last 3 months?	4 (3.7)
Has your patient been more fatigued in the last 3 months?	46 (43)
Has your patient experienced increased mobility difficulties in the last 3 months?	21 (19.6)
Has your patient complained of memory problems?	7 (6.5)
Does your patient present slow gait speed (i.e., >4 s to walk 4 m)?	17 (15.9)
GFST, m (sd)	1.2 (1.2)
Do you think your patient is frail?	
No	70 (65.4)
Yes	37 (34.6)

**Table 4:** Frailty measured by sZFS.

	*n* = 107
sZFS items	
Does the person live alone at home?	34 (31.8)
Is there a weight loss greater than or equal to 5% in 6 months?	4 (3.7)
Monopod support test <5 s?	26 (24.3)
Does the person complain of memory problems?	7 (6.5)
Does the person have prescriptions for more than 5 therapeutic classes on his/her prescription history for less than 6 months?	41 (38.3)
sZFS, m (sd)	1 (1.1)
Classification	
Not frail	42 (39.3)
Pre-frail	51 (47.7)
Frail	14 (13.1)
Frailty according to sZFS	
No	42 (39.3)
Yes	65 (60.7)

**Table 5:** Pearson correlation between the items of the two tools: “sZFS” and GFST’s criteria.

Pearson Correlation												
		GFST						sZFS				
		Alone at Home	Weight Loss in the Last 3 Months	Fatigue	Increased Mobility Difficulties	Memory Problems	Slow Gait Speed	Alone at Home	Weight Loss Greater than or Equal to 5% in 6 Months	Monopod Support	Memory Problems	More than 5 Therapeutic Classes
GFST	Alone at home	1	−0.13	0.26	0.17	0.14	0.25	1	−0.13	0.27	0.14	0.45
	Weight loss in the last 3 months	−0.13	1	0.13	0.15	−0.05	0.05	−0.13	1	0	−0.05	−0.05
	Fatigue	0.26	0.13	1	0.14	−0.08	0.09	0.26	0.13	0.21	−0.08	0.25
	Increased mobility difficulties	0.17	0.15	0.14	1	−0.04	0.43	0.17	0.15	0.43	−0.04	0.14
	Memory problems	0.14	−0.05	−0.08	−0.04	1	−0.01	0.14	−0.05	−0.06	1	0.26
	Slow gait speed	0.25	0.05	0.09	0.43	−0.01	1	0.25	0.05	0.59	−0.01	0.34
sZFS	Alone at home	1	−0.13	0.26	0.17	0.14	0.25	1	−0.13	0.27	0.14	0.45
	Weight loss greater than or equal to 5% in 6 months	−0.13	1	0.13	0.15	−0.05	0.05	−0.13	1	0	−0.05	−0.05
	Monopod support	0.27	0	0.21	0.43	−0.06	0.59	0.27	0	1	−0.06	0.09
	Memory problems	0.14	−0.05	−0.08	−0.04	1	−0.01	0.14	−0.05	−0.06	1	0.26
	More than 5 therapeutic classes	0.45	−0.05	0.25	0.14	0.26	0.34	0.45	−0.05	0.09	0.26	1

sZFS’s scale therefore appears to be well constructed, with very little redundancy.

**Table 6:** Contingency table–Zulfiqar frailty scale vs. GFST’s criteria, with “pre-frail” and “robust” patients making up the “non-frail” group.

		GFST	
		Frail	Not frail
sZFS	Frail	37	28
	Not frail	3	39

Interpretation: The sZFS has good sensitivity and NPV.

**Table 7:** Scales administration time.

	Duration Time, m (sd)	CI 95%
GFST	87 (22)	[83; 91]
sZFS	77 (19)	[74; 81]

## Data Availability

The datasets used and/or analyzed during the current study are available from the corresponding author on reasonable request.
